# Measurement of Time Taken by the Formosan Termite, *Coptotermes formosanus*, to Pass Tunnel Intersections

**DOI:** 10.1673/031.012.2301

**Published:** 2012-02-09

**Authors:** Sook Jung Ku, Su Nan-Yao, Lee Sang-Hee

**Affiliations:** ^1^ Division of Forest Resources, College of Forest and Environmental Sciences, Kangwon National University, Kangwon, South Korea; ^2^ Department of Entomology and Nematology, Ft. Lauderdale Research and Education Center, University of Florida, Ft. Lauderdale, Florida, USA; ^3^ Division of Fusion Convergence of Mathematical Sciences, National Institute for Mathematical Sciences, Daejeon, South Korea

**Keywords:** foraging efficiency, termite tunnel intersection, termite tunnel network, traffic efficiency

## Abstract

Subterranean termites build complex tunnel networks below ground for foraging. During the foraging activity, termites may encounter a considerable number of tunnel intersections. When they encounter the intersections, they spend some time gathering information for making a decision regarding their moving direction by anntenation. The spent time is likely to be directly connected to the termites' survival because depending on the time, the total traveling time taken by the termites for transferring food resources from the site of food to their nest can vary significantly because of many intersections. In the present study, we measured the time spent by a termite to pass an intersection with widths of *W_1_* and *W_2_* (*W_1_* and *W_2_*: 2, 3, or 4 mm); *τ_L_*, *τ_R_*, and *τ_s_* are the passing time for turning left, turning right, and going straight, respectively. *W_1_* represents the width of the tunnel in which the termites advanced, and *W_2_* represents the width of the other tunnel encountered by the advancing termites. For the combinations of *W_1_* and *W_2_,* (*W_1_, W_2_*) = (2, 2), (3, 3), (2, 3), (2, 4), (3, 4), and (4, 3), the values of *Tτ_L_, τ_R_,* and *τ_s_* in each case were statistically equal. For (*W_1_, W_2_*) = (3, 2), (4, 2), and (4, 4), *τ_s_* was shorter than *τ_R_* and *τ_R_* in each case. The experimental results are briefly discussed in relation to the termite foraging efficiency.

## Introduction

Subterranean termites forage for food resources by constructing tunnel networks below ground. The network geometry reflects a compromise between the foraging efficiency and other biological and/or ecological constraints such as the number of active foragers, soil density, and food availability (Le Comber et al. 2006; Lee et al. 2007a). Hence, understanding the tunnel network pattern may provide one important key to understanding the termite foraging behavior towards maximizing the foraging efficiency (Campora and Grace 2001; Puche and Su 2001). With simulation studies, Lee et al. (2006, 2007, 2009), Lee and Su (2009a, 2009b 2010), and Jeon et al. (2010) reported the relationship between tunnel network patterns and foraging efficiency. Thus far, most theoretical research on how a termite tunnel pattern is efficient in foraging has concentrated on the geometry of the tunnel patterns at a colony level.

In contrast, two papers (Lee et al. 2008; Ku et al. 2010) dealt with termite behavior in relation to the foraging efficiency at an individual level. Lee et al. (2008) revealed that when termites passed a corner of a bent tunnel, they moved faster in a rounded corner than in a sharp corner, which in turn improved the termite tunnel—traffic efficiency. The study of Lee et al. (2008) revealed that the traffic efficiency, defined as the termite moving speed in tunnels, is an important factor affecting the foraging efficiency. Ku et al. (2010) showed that when termites encounter tunnel intersections while traveling in the tunnel network, they selected a relatively wide tunnel. The wide paths are likely to be paths that termites more traveled, which would indicate economic paths to decrease their traveling distance from the food resources to their nest.

In the present study, as a follow—up research to that of Ku et al. (2010), we measured the time required for a termite to pass the intersection with combinations of widths (*W_1_* and *W_2_*: 2, 3, or 4 mm) while selecting different directions-turning left, turning right, or moving straight. The passing time could be an important factor affecting the foraging efficiency because while traveling in a tunnel network, termites are likely to encounter numerous tunnel intersections. A small time difference in selecting a different direction could accumulate to a large value of the total traveling expenses.

## Materials and Methods

Formosan subterranean termites, *Coptotermes formosanus* Shiraki (Isoptera: Rhinotermitidae), were collected from monitoring stations by using the method of Su and Scheffrahn (1986). The collected termites were brought back to the laboratory and were separated from their stations and processed immediately in the laboratory as described by Tamashiro et al. (1973). Termites were then placed in a release chamber that contained wooden sticks (length: approximately 3 cm) as a food source. The chamber was kept at 27 ± 2 °C.

Two—dimensional foraging arenas were used for this study. The experimental arena consisted of two layers (13 × 13 cm) of clear Plexiglas (thickness: 2 mm) and a middle layer. The middle layer had a circular space (diameter: 10 cm; height: 2 mm). A hole (1.5 cm diameter) was drilled on the top and middle layers of the arena to allow termite entry. The 2 mm gap between the Plexiglas sheets were filled with sifted sand (0.3–0.35 mm, sieved) moistened with deionized water (approximately 7% sand by weight). The sand in the middle layer was compacted to a bulk density of approximately 1.3 g/cm^3^.

**Figure 1.**
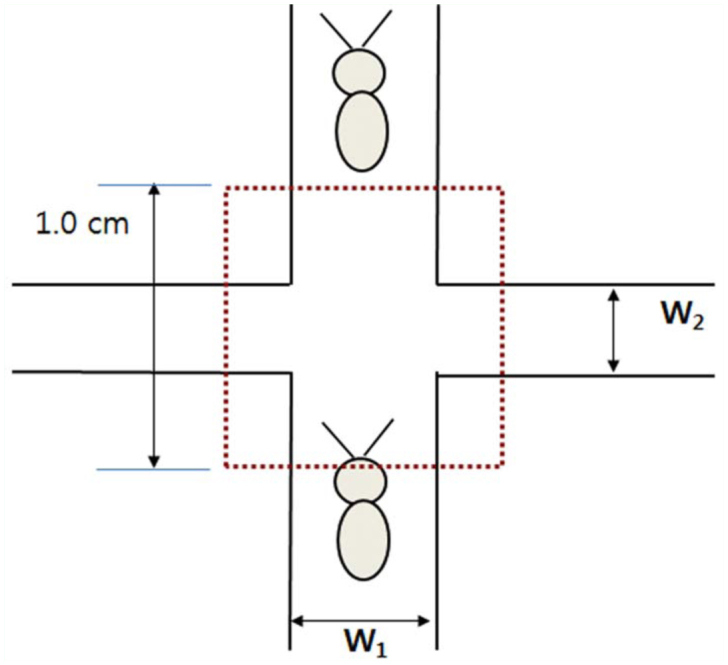
Schematic representation of tunnel intersection with two tunnel widths, *W_1_* and W_2_. W_1_ represents the width of a tunnel in which a termite advances, and *W_2_* is the width of the other tunnel. The red box represents the tunnel intersection defined in this study. High quality figures are available online.

After opening the top layer, two tunnels intersecting at a 90° angle in the sand substrate were carefully excavated using a sharp cutting knife which took less than one minute. As soon as the excavation was complete, the top layer was covered to minimize moisture evaporation. The ends of the two tunnels were connected to the introduction holes, and the tunnels had varying widths of *W_1_* and *W_2_* (i.e., 2, 3, or 4 mm), where *W_1_* represents the width of the tunnel in which the termites advanced, and *W_2_* represents the width of the other tunnel encountered by the advancing termites (Figure 1).

Three to eight replications were carried out for each combination of *W_1_* and *W_2_* (*W_1_, W_2_*). Each arena was placed in a horizontal position in a room kept at 26 °C. Forty workers plus 4 soldier termites were introduced into the arena through the introduction hole. The holes were then covered with plastic plugs to prevent airflow. The 44 termites were allowed to acclimatize to their new environment for 20 min. After this period, their movements were continuously recorded for one hour with a digital camcorder mounted above the substrate. Using the recording, we measured the time, τ, taken by a termite to pass the intersections and select different directions, i.e., turning left (*τ_L_*), turning right (*τ_R_*), or moving straight (*τ_S_*). The intersection area was defined as a 10 × 10 mm square (Figure 1). Only data of worker termites that were physically uninfluenced by the other termites at the tunnel intersection were used for analysis.

## Results

As soon as the termites were introduced into the experimental area with a preformed tunnel intersection, they began to move along the tunnel. This behavior is likely to be escaping behavior. Thus, 10 minutes were given for termites to adapt to the arena. After the time, termite movement behavior was observed at the intersection. Some termites stopped walking and antennated laterally to touch the tunnel wall for a short period of time presumably to obtain information of the sudden change in the circumambient geometry (Lee et al. 2008), but others kept moving straight or attempted to change their moving direction without the antennation behavior. Other additional behaviors were observed. For example, some termites determined their direction at the intersection and turned their body to pass the intersection. After that, they stopped moving and began to move back to the intersection. Such kind of behavior was excluded from analysis because it occurred irregularly and difficult to characterize.

**Table 1.**
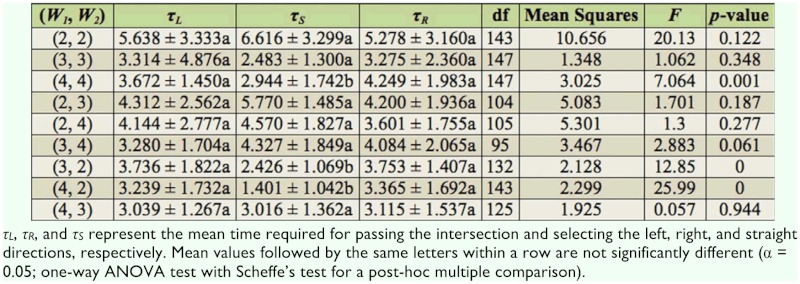
Mean time (seconds) ± SD for a termite to pass at the intersection of tunnels with widths of *W_1_* (mm) and *W_2_* (mm) in nine arenas.

**Figure 2.**
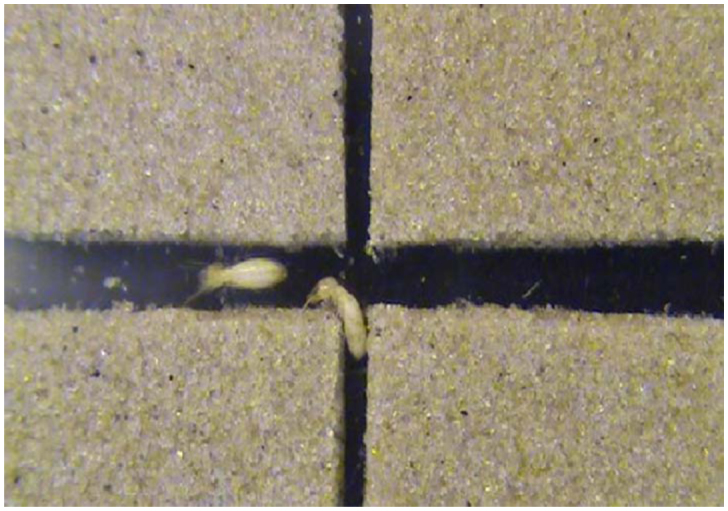
Behavior of a termite, turning its body vertically, while changing its moving direction at the intersection with the values of (2, 4). High quality figures are available online.

**Figure 3.**
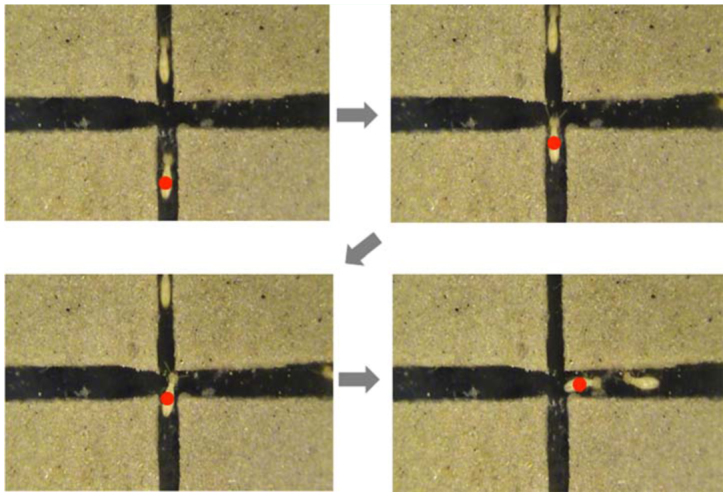
Photographs showing that an advancing termite is likely to be confronted with the corner of the intersection when it changes its moving direction. High quality figures are available online.

For arenas in which (*W_1_, W_2_*) = (2, 2) and (3, 3), the values of *τL, τ_R_,* and *τ_s_* in each case were statistically the same (see Table 1). This was because the selection of different directions provided no advantage with respect to the tunnel width, and in particular, the narrow intersection area in comparison to the termite body length (∼3 mm) made walking difficult, which in turn diluted the time difference in selecting different directions. However, for (*W_1_, W_2_*) = (4, 4), the value of *τ_S_*was significantly smaller than the values of *τ_L_*and *τ_R_,* while the values of *τ_L_* and *τ_R_* were statistically the same in each case. Most termites moving straight passed the intersection without stopping. In the case that termites advanced from a relatively narrow tunnel to the intersection connecting to wider tunnels ((*W_1_, W_2_*) = (2, 3), (2, 4), and (3, 4)), the values of *τ_L_, τ_R_,* and *τ_s_* were statistically the same in each case. For (*W_1_, W_2_*) = (2, 3) and (2, 4), turning into wider tunnels at left or right may have saved time (hence *τ_L_, τ_R_* < *τ_s_*), but termites also took more time to change their direction, and during the direction change at the intersection they turned their body vertically and walked on the sidewall (Figure 2). This behavior increased *τ_L_* and *τ_R_,* which consequently led to the result *τ_L_* = *τ_R_* = *τ_S_.* For (*W_1_, W_2_*) = (3, 4), many termites turning left or right touched the right—angled corner of the intersection because of the deviation in their walking (Figure 3). As soon as the termites touched the corner, they exhibited the anntenation behavior. This diluted the advantage of the relatively large tunnel width, which consequently led to the statistically equal values of *τ_L_, τ_R_,* and *τ_s_.* When the termites advanced from a relatively wide tunnel to the intersection connecting to narrower tunnels ((*W_1_, W_2_*) = (3, 2) and (4, 2)), the value of *τ_s_* was significantly smaller than the values of *τ_L_* and *τ_R_* because of the same reason as the case of (*W_1_, W_2_*) = (4, 4).

## Discussion

Previous studies (Lee et al. 2006, 2007a, 2007b, 2009a, 2009b) showed that the geometry of a termite tunnel network is closely related to foraging efficiency at the colony level. Further studies revealed that the traffic efficiency (defined as the termite's walking speed in tunnels) and the directional selection at the tunnel intersections were important factors affecting the foraging efficiency (Lee et al. 2008; Ku et al. 2010).

During foraging activity, termites encounter numerous tunnel intersections. Termites preferred to select a relatively wide tunnel at the intersections, which could be a mechanism to find the shortest path from the food resources to the nest. In the present study, we measured the time taken by a termite to pass an intersection and selecting a different direction—turning left, turning right, and moving straight. This information is important to understand termite foraging behavior in maximizing foraging efficiency. Animal foraging efficiency is determined in large part by food search and transport efficiency (Torres-Contreras and Vasquez 2004; Pyke 1978). Conceptually, the foraging efficiency for termites can be defined as the ratio of the number of encountered food resources for foraging time to the sum of the shortest length from the location of the food to the nest (Lee et al. 2007a). Thus, the measured time for each direction is directly related to the foraging efficiency because the time could be accumulated during the termites' travel in the tunnel networks. Termites spent significantly less time to pass an intersection in the case of (*W_1_, W_2_*) = (3, 2), (4, 2), and (4, 4). In the other cases, no difference in the passing time was found. When traveling in the tunnel network, termites are likely to be faced with two intersection types: *W_1_* < *W_2_* or *W_1_* > *W_2_,* where *W_1_* is the width of the tunnel where termites are present, and *W_2_* is the width of the connecting tunnel termites may encounter. Most advancing termites faced with the case of *W_1_* < *W_2_* would change their moving direction at the intersection, following the relatively wide tunnel selection mechanism; subsequently, they would confront the case of *W1* > *W_2_.* For an established colony, most termites in a tunnel network may travel along the relatively wide tunnels, and our results showed that termites advancing from a relatively wide tunnel into the intersection may continue going straight, which could be a viable strategy for improving the foraging efficiency. It is unknown, however, if our results are consistent with field observations because there could be many other constraining conditions associated with the physical factors such as soil hydrology and soil particle size (Su and Puche 2003). In addition to these factors, the various widths and wall roughness of a natural termite tunnels may affect the time taken by a termite to pass an intersection (Lee et al. 2008a, 2008b). Additionally, in this study, we simplified the tunnel intersections as two linear tunnels intersecting at 90°, which may be valid at the scale of termite body length (3–4 mm). Su et al. (2004) reported that the intersection angles are in the range of ∼70–90° in many cases, which support the validation of the simplification. However, for some cases we found intersection angles measuring less than 50°. Thus, the effect of the intersection angles needs to be considered. Nevertheless, results obtained in this study are valuable because they provide insights into the foraging efficiency at the individual level. These results also suggest directions for future empirical investigations of the termite foraging strategy in relation to the traffic efficiency.
